# King Edward's Hospital Fund for London

**Published:** 1903-12-19

**Authors:** 


### HOSPITAL ADMINISTRATION.

### CONSTRUCTION AND ECONOMICS.

### KING EDWARD'S HOSPITAL FUND FOR LONDON.

#### DISTRIBUTION MEETING AT MARLBOROUGH HOUSE.

A meeting of the General Council of this Fund for the purpose of awarding grants to the hospitals for the present year was held on the 14th inst. at Marlborough House, H.R.H. the Prince of Wales being in the chair. Those present were:—The Duke of Fife, the Bishop of London, the Rev. J. Guinness Rogers, D.D., the Chief Rabbi (the Rev. Dr. Adler), Viscount Duncannon, Lord Rothschild, Lord Iveagh, Lord Farquhar, the President of the Hospital Sunday and Hospital Saturday Funds (the Lord Mayor), the Chairman of the County Council (Lord Monkswell), the President of the Royal College of Physicians (Sir William Church, Bart.), the Rt. Hon. Sir Savile Crossley, Bart., M.P., the Rt. Hon. Sir Joseph Dimsdale, Bart., M.P., the Rt. Hon. C. Stuart Wortley, K.C., M.P., Sir John Aird, Bart., M.P., Sir Henry Burdett, K.C.B., Sir W. J. Collins, Sir H. G. Howse, Sir J. G. Craggs, Mr. Sydney Buxton, M.P., Mr. F. M. Fry, Mr. William Latham, K C., Mr. J. Pierpont Morgan, Jun., Mr. Albert G. Sandeman, Mr. H. C. Smith.

Letters of regret were read from the following:—The Duke of Bedford, Archbishop Bourne, the Rev. Dr. Bowman Stephenson, D.D., Lord Strathcona and Mount Royal, Sir Trevor Lawrence, Bart., Mr. Thomas Burt, M.P., and Mr. Julius Wernher. The Minutes of the last meeting having been read and confirmed, the hon. treasurer, Lord Rothschild, in presenting the accounts said: Your Royal Highness, it is my humble duty now to present to you the accounts of the Hospital Fund as they stand at the present moment. The year is not yet ended, so that the accounts now presented to you are incomplete, but I express the hope that before the year is over we shall receive some substantial sums, and although I do not suppose we shall be in the position we were in last year, still there will be large amounts coming in to increase the sums your Royal Highness has to dispose of. The figures, I see from the present note, do not include a contribution from the League of Mercy, but I understand we shall receive a larger sum from that society than the £8,000 received last year. There are also donations and subscriptions that generally come in at the end of the year. The amount received to the 10th instant was £71,033 8s. 3d. I think the subscribers to this Fund ought to know and to bear in mind that this large sum of money which does such an extraordinary amount of good, not only by its distribution, but by the example given, is collected and divided at a cost of under £2,000 a year. This, I think, speaks very highly for our honorary officials. I do not know that it is necessary on this occasion for me to say anything more, except to express a hope that before the end of the year your Royal Highness may receive a considerable addition to this Fund, and that many others may be induced by your influence to subscribe largely. (Hear, hear.)

Sir H. Burdett said that he was glad to inform the meeting that the League of Mercy would be able to send a contribution of £10,000 to the Fund, which was £2,000 more than last year, and ten times as much as the amount given in the first year of the establishment of the League. The Chairman of the Executive Committee (Mr. H. C. Smith) presented the Report of the Executive Committee as follows:—

The Executive Committee recommend this year the distribution of £99,000 to the London hospitals, making, with the £1,000 entrusted to the Fund by the London Parochial Charities for the Convalescent Homes, a total distribution of £100,000.

This course, which involves the withdrawal of a substantial sum from the moneys placed at the discretion of the Council, has not been adopted without the most grave reflection.

It was considered, however, that such a step should be taken in view of the fact that the hospitals are urgently in needs of funds this year, and that the efforts of the Fund for the present should, in the opinion of this Committee, be devoted to meeting the pressing needs of the present generation.

The Committee feel assured that in this decision, taken with the approval of H.R.H. the President, they will have the support of the subscribers generally and of all those in London who are interested in hospital work.

They hope that during the year approaching increased support may enable them to replace the sum thus taken from reserve.

The Chairman of the Hospitals Distribution Committee (Sir William Church) presented the report as follows:—

The Committee desire to record the great loss they have sustained in the retirement of Lord Lister from his position as their Chairman, the arduous and responsible duties of which he had so well performed ever since the Fund was founded. They desire to express their deep appreciation of the services he has rendered and their regret that his advancing years and the state of his health compelled him reluctantly to retire.

Sir William Church was elected Chairman in his stead.

The Committee have the pleasure to report that the Executive Committee, with the approval of his Royal Highness the President, placed in their hands this year the sum of £99,000 for distribution among the London hospitals.

The advances of medical science entail great expense on existing hospitals to bring the condition of their buildings and equipment up to the standard of modern requirements, thus causing a great drain on the funds supplied by the benevolence of the charitable public. At the same time the necessary expenditure on patients tends to increase, so that the needs of the hospitals are more urgent than ever.

Now that with the aid of his Majesty's Fund nearly all the closed beds in the metropolitan institutions have been reopened, the Committee considered that their efforts should be directed to assisting, as far as their funds permitted, the development of hospitals in localities where accommodation was insufficient.

With the exception of the grant to the Poplar Hospital, which is given to help in opening ten more beds for the use of the sick poor, no further sums have been allocated under this heading.

The numbers of beds reopened in the different years are as follows:—

| | |
|---|---|
| 1897 | 185 |
| 1898 | 57 |
| 1899 | 45 |
| 1900 | — |
| 1901 | 34 |
| 1902 | 112 |
| 1903 | 10 |
| Total beds reopened | 443 |

These have been reopened as free beds.

The Committee are of opinion that one first-rate orthopædic hospital, with its country branch, will tend more to the general benefit of this class of patient than a number of institutions in London in rivalry with one another. They regret that the City Orthopædic has not at present seen its way to join in any scheme for amalgamation. The Committee consider that, although the sum set aside for the amalgamation scheme as stated below is large it will be money well spent.

The Committee further recommend that, in accordance with the resolution passed by the General Council of the Fund last year, other sums should from time to time be set aside with a view to the possible amalgamation of other hospitals.

A large grant has also been recommended to King's College Hospital, the removal of which to the South of London they view with the greatest satisfaction, and feel assured that the supporters of the Fund will appreciate the importance of encouraging the removal of this hospital.

Subscribers to the Fund will note the various conditions on which grants are made. In addition to remarks published with regard to certain hospitals, it shoutd be mentioned that private letters are in many instances written to individual hospitals containing various suggestions.

The visitors' reports on the various hospitals have been of the greatest advantage to the Committee, who wish to express their appreciation of the services thus rendered.

The details of the awards to the various hospitals are appended.

Sir Savile Crossley read the awards as follows:—

### THE KING'S HOSPITAL FUND, 1903.

### LIST OF GRANTS TO THE HOSPITALS.

Alexandra Hospital for Children, Queen Square, Bloomsbury, W.C.—£500 donation.

Belgrave Hospital, Clapham Road, Kennington.—£750 donation to building.

Blackheath and Charlton Cottage Hospital, Shooter's Hill Road, Blackheath, S.E.—£50 donation to equipment of operating room.

Bolingbroke Hospital, Wandsworth Common, S.W.—£1,000 donation, £500 to improvements.

British Lying-in Hospital, Endell Street, St. Giles, W.C.—£250 donation.

Cancer Hospital, Fulham Road, Brompton, S.W.—£200 donation.

Central London Ophthalmic Hospital, 238*a* Gray's Inn Road, W.C.—£200 donation.

Central London Throat and Ear Hospital, Gray's Inn Road, W.C.—£200 donation.

Charing Cross Hospital, Agar Street, West Strand, W.C.—£2,000 donation towards rebuilding fund.

Chelsea Hospital for Women, Fulham Road, S.W.—£350 donation.

Cheyne Hospital, Cheyne Walk, Chelsea, S.W.—£100 donation in consideration of the fact that curable cases are admitted.

City of London Hospital for Diseases of the Chest, Victoria Park, E.—£1,500 annual to support beds reopened by this Fund; £500 donation.

City Orthopædic Hospital, 26 and 27 Hatton Garden, E.C.—£250 annual to maintain beds re-opened by this Fund; committee regret that this hospital has not yet consented to the proposed amalgamation.

Clapham Maternity Hospital, 41 and 43 Jeffreys Road, Clapham, S.W.—£100 donation.

"Dreadnought" Hospital, Greenwich, S.E.—£500 annual for general purposes, £1,000 for Tropical Diseases Department; £1,500 donation.

East End Mothers' Home, 394 and 396 Commercial Road, E.—£200 donation.

East London Hospital for Children, Glamis Road, Shadwell, E.—£1,600 donation, provided isolation and whooping cough wards are put in hand in 1904.

Eltham and Mottingham Cottage Hospital, High Street, Eltham.—£50 donation.

Establishment for Gentlewomen, 90 Harley Street, W.—£250 donation.

Evelina Hospital, Southwark Bridge Road, S.E.—£250 donation, to cost of out-patient department.

French Hospital, 172 Shaftesbury Avenue, W.C.—£100 donation.

Friedenheim Hospital, Sunnyside, Upper Avenue Road, Swiss Cottage, N.W.—An excellent institution.

General Lying in Hospital, York Road, Lambeth, S.E.—£200 donation.

German Hospital, Dalston Lane, N.E.—£150 donation.

Gordon Fistula Hospital, Vauxhall Bridge Road, S.W.—£50 donation.

Great Northern Central Hospital, Holloway Road, N.—£750 annual to maintain beds re-opened by this Fund; £750 donation.

Grosvenor Hospital for Women and Children, Vincent Square, Westminster, S.W.—£200 donation.

Guy's Hospital, St. Thomas Street, Borough, S.E.—£5,000 annual to maintain beds reopened by this Fund; £1,000 donation.

Hampstead Hospital, Parliament Hill, N.W.—£1,000 donation to building fund.

Home and Infirmary for Sick Children, Sydenham Road, Lower Sydenham, S.E.—£100 donation.

Hospital for Consumption, Brompton, S.W.—£1,000 donation to country branch.

Hospital for Diseases of the Throat, Golden Square, W.—£100 donation towards fire apparatus.

Hospital for Epilepsy and Paralysis, 4 Maida Vale, W.—£350 donation.

Hospital for Sick Children, Great Ormond Street, Bloomsbury, W.C.—£500 annual to maintain beds reopened by this Fund; £750 donation.

Hospital for Women, Soho Square, W.—£1,100 annual to maintain beds repened by this Fund.

Hospital of St. John and St. Elizabeth, Grove End Road, St. John's Wood.—£500 annual to maintain beds re-opened by this Fund.

Italian Hospital, Queen's Square, Bloomsbury, W.C.—£500 annual to maintain beds reopened by this Fund.

Kensington Dispensary and Children's Hospital, 49 Church Street, Kensington, W.—Not eligible as a hospital on present information.

King's College Hospital, Portugal Street, Lincoln's Inn, W.C.—£1,000 annual to meet deficiency, £1,500 for general maintenance, £5,000 to the fund for removal of the hospital to South London which the Committee view with great satisfaction; £6,500 donation.

London Fever Hospital, Liverpool Road, Islington, N.—£600 donation.

London Homœopathic Hospital, Great Ormond Street, W.C.—£200 donation.

London Hospital, Whitechapel Road, E.—£5,000 annual to meet expenditure out of capital made necessary by improvements in the wards; £2,000 donation.

London Lock Hospital (Female), Harrow Road, W.—£600 donation.

London Temperance Hospital, Hampstead Road, N.W.—£500 donation for out-patients' department, conditional on the work being put in hand during 1904.

London Throat Hospital, 204 Great Portland Street, W.—£50 donation,

Medical Mission Hospital (formerly entered as Cottage Hospital, Canning Town), 538, 540 Barking Road, Plaistow.—£350 donation; £250 to building.

Memorial Cottage Hospital, Mildmay Park, N.—£50 donation.

Metropolitan Hospital, Kingsland Road, N.E.—£500

annual to maintain beds reopened by this Fund; £2,000 donation.

Middlesex Hospital, Mortimer Street, W.—£1,000 annual to meet deficiency. The committee note with satisfaction abolition of the practice of keeping butter in the lockers by beds; £1,500 donation.

Mildmay Mission Hospital, Austin Street, Bethnal Green, E.—£250 donation.

Miller Hospital, Greenwich Road, S.E.—£1,000 donation to building.

Mount Vernon Hospital for Consumption (formerly entered as North London Hospital for Consumption), Mount Vernon, Hampstead, N.W.—£1,000 annual to support beds reopened by this Fund; £500 donation.

National Dental Hospital, 187, 191 Great Portland Street, W.—£50 donation.

National Hospital for Diseases of the Heart, 32 Soho Square, W.—£50 donation towards $x$-ray apparatus.

National Hospital for the Paralysed and Epileptic, Queen Square, Bloomsbury, W.C.—£750 annual to meet deficiency, £500 to improvements; £750 donation.

National Orthopædic Hospital, 234 Great Portland Street, W.—£400 donation. Committee view with satisfaction the amalgamation with the Royal Orthopædic Hospital.

New Hospital for Women, 144 Euston Road, N.W.—£300 donation.

North-Eastern Hospital for Children, Hackney Road, Shoreditch, N.E.—£500 annual towards maintenance, £1,000 for building; £1,000 donation.

North-West London Hospital, 18–22 Kentish Town Road, N.W.—£750 donation. Committee urge the rebuilding of this hospital.

Norwood Cottage Hospital, Hermitage Road, Central Hill, Upper Norwood, S.E.—£50 donation.

Oxygen Hospital, 2 Fitzroy Square, W.

Paddington Green Children's Hospital, Paddington Green, W.—£400 donation.

Passmore Edwards' Acton Cottage Hospital, Gunnersbury Lane, Acton, W.—Committee are glad to say that it does not require help from the Fund this year.

Passmore Edwards' Hospital, Shrewsbury Road, East Ham.—£50 donation.

Passmore Edwards' Hospital for Willesden, Harlesden Road, N.W.—Committee are glad to say that it does not require help from the Fund this year.

Passmore Edwards' Cottage Hospital, Bounds Green Road, Wood Green, N.—£50 donation.

Poplar Hospital, Blackwall, E.—£500 annual to help to open closed beds.

Queen Charlotte's Lying-in Hospital, Marylebone Road, N.W.—£500 donation.

Queen's Jubilee Hospital, Richmond Road, Earl's Court, S.W.—£200 donation to building fund. Committee consider a new hospital urgently required in this district.

Royal Dental Hospital of London (formerly entered as Dental Hospital), Leicester Square, W.C.—£300 donation to debt on building.

Royal Ear Hospital, Frith Street, Soho Square, W.—No grant.

Royal Eye Hospital, St. George's Circus, Southwark, S.E.—£250 donation.

Royal Free Hospital, Gray's Inn Road, W.C.—£750 annual to meet deficiency, £1,500 towards improvements; £2,000 donation.

Royal Hospital for Children and Women, Waterloo Bridge Road, S.E.—£1,000 donation for building.

Royal Hospital for Diseases of the Chest, 231 City Road, E.C.—£500 donation towards the open-air sanatorium.

Royal London Ophthalmic Hospital, City Road, E.C.—£2,000 annual; £2,500 donation to help to maintain the beds opened by this Fund; Committee consider this hospital should receive more support from the public.

Royal Orthopædic Hospital, 297 Oxford Street, W.—£200 donation. Committee view with satisfaction the amalgamation with the National Orthopædic Hospital.

Royal Westminster Ophthalmic Hospital, King William Street, West Strand, W.C.—£400 donation to debt.

St. George's Hospital, Hyde Park Corner, S.W.—£2,000 donation.

St. John's Hospital for Diseases of the Skin, Out-Patient Department, 49 Leicester Square, W.C; In-patient Department, 238 Uxbridge Road, W.—£50 donation to Shepherd's Bush Branch.

St. John's Hospital, Morden Hill, Lewisham, S.W.—£100 donation.

St. Mark's Hospital, City Road, E.C.—£100 donation.

St. Mary's Children's Hospital, Plaistow, E.—£600 donation.

St. Mary's Hospital, Praed Street, Paddington, W.—£1,000 annual to meet deficiency. £1,000 donation.

St. Monica's Hospital, Brondesbury Park, N.W.—£50 donation.

St. Peter's Hospital for Stone, Henrietta Street, Covent Garden, W.C.—£100 donation.

St. Saviour's Hospital, Osnaburgh Street, Regent's Park, N.W.—£100 donation.

St. Thomas's Hospital, Albert Embankment, S.E.—£1,800 donation to maintain the beds reopened.

Salvation Army Maternity Hospital, Ivy House, 271 Mare Street, Hackney, N.E.—£50 donation.

Samaritan Free Hospital, Marylebone Road, N.W.—£500 annual towards maintenance. £200 to New out-patients' department. £200 donation.

Santa Claus Home, Cholmeley Park, Highgate, N.—£50 donation in consideration of the fact that curable cases are admitted.

South Wimbledon and Merton Cottage Hospital, 145 Merton Road, Wimbledon.—£50 donation for operative appliances.

Tottenham Hospital, Tottenham, N.—£1,000 annual to maintain beds reopened by this fund. £2,000 to building. £2,000 donation.

University College Hospital, Gower Street, W.C.—£1,000 annual to support beds reopened by this Fund; £2,000 donation.

Victoria Hospital for Sick Children, Tite Street, Chelsea, S.W.—£800 donation.

West End Hospital for Diseases of the Nervous System, 73 Welbeck Street, W.—£150 donation towards isolation ward.

Western Ophthalmic Hospital, 153 and 155 Marylebone Road, W.—No grant. To be reconsidered when proper progress has been made towards collecting the rebuilding fund.

West Ham Hospital, Stratford, E.—£300 annual towards maintenance; £2,000 donation to building.

West London Hospital, Hammersmith Road, W.—£1,500 annual to maintain beds reopened by this Fund; £1,000 donation conditional on the bath-rooms, etc., on east side being put in hand at once.

Westminster Hospital, Broad Sanctuary, S.W.—£750 annual towards deficiency; £1,000 donation.

Woolwich and Plumstead Cottage Hospital, Shooter's Hill, Kent.—£50 donation.

Special grant set aside to be drawn on as required for amalgamation of hospitals, £10,500.

Mr. Wm. Latham, K.C., read the following report of the Convalescent Homes Committee.

Owing to the continued liberality of the grant made by the trustees of the London Parochial Charities the committee have again £1,000 to distribute.

The committee have again followed the course adopted in previous years of making out of the limited sum at their disposal fairly substantial grants to a comparatively small number of institutions, and to a great extent they have repeated grants to those before approved of, in preference to varying the distribution.

The following awards were recommended :—

Alexandra Hospital, Convalescent Home at Painswick.—£50.

Charing Cross Hospital, Convalescent Home at Limpsfield, Surrey.—£50.

East London Hospital for Children, Convalescent Home at Bognor.—£100.

King's College Hospital, Convalescent Home at Hemel Hempsted.—£100.

Mary Wardell Convalescent Home, Stanmore, Middlesex.—£50.

Metropolitan Hospital, Convalescent Home at Cranbrook.—£25.

Metropolitan Convalescent Institution, 32 Sackville Street, Piccadilly, W.—£200.

Middlesex Hospital, Convalescent Home at Clacton-on-Sea.—£100.

Mrs. Kitto's Convalescent Home, South Park, Reigate. £25.

National Hospital for the Paralysed and Epileptic, Convalescent Home at East Finchley.—£50.

Paddington Green Children's Hospital, Convalescent Home at Slough.—£50.

Queen Charlotte's Lying-in Hospital, Convalescent Home at 20 Victoria Road, Kilburn.—£50.

St. George's Hospital and Atkinson Morley's Convalescent Hospital at Wimbledon.—£50.

St. Mary's Hospital for Sick Children, Convalescent Home at Southend-on-Sea.—£50.

Victoria Hospital, Convalescent Home at Broadstairs. £50.

Total grants to Convalescent Homes.—£1,000.

His Royal Highness, in moving the adoption of the report of the executive committee, said :—

My Lords and Gentlemen : I beg to move the adoption of the report, and in doing so to express my hearty concurrence with the recommendations of my committees, and approval of the various grants, and of the conditions upon which in some instances these have been made.

You will I know join with my feelings of deep regret at our loss through the deaths of Cardinal Vaughan and Lord Rowton. We have also sustained a further serious loss in the retirement of Lord Lister who, since the inception of the Fund, has been chairman of the distribution committee. I trust that with improvement in his health we may again have the benefit of his valuable services.

In reviewing the results of this year's work I have to admit that the hopes which I expressed twelve months ago, that another £10,000 a year would be forthcoming, have not been fulfilled. But the year 1902 was an exceptional one, as a large sum was contributed specially as a Coronation gift.

We have, however, in addition to the sums set aside to be invested and those given in the form of investments, a considerable balance which can be utilised at the discretion of the General Council. Upon this balance we have decided to draw for this year only, in order that the amount for distribution among the hospitals may not be less than that of last year.

We have not come to this decision without duly considering those two opposing principles, one of which deprecates any encroachment upon our reserves, while the other advocates the expenditure of subscriptions with a view of relieving present necessity rather than of placing them to capital account.

We are anxious not to go back in the work initiated by the King, and in the fulfilment of which I consider a sacred trust was confided to me. Our aim must be steady progress and to secure public confidence.

I venture to draw attention to the enormous sums for which appeals are now contemplated by the London hospitals. According to my information, if we take only 12 hospitals out of more than 100, thus omitting many which are contemplating appeals, these sums amount to £700,000. This is exclusive of a probable appeal on behalf of St. Bartholomew's Hospital for a sum varying between £300,000 and £600,000. Hence the public must be prepared for appeals to provide over a million ! And in addition to this there are other hospitals not included in this list, many of which have equally pressing needs.

It is, indeed, a serious question whether some limit should not be put upon hospital extension works, and I trust that new schemes will not be undertaken by any hospitals without grave consideration of these heavy demands now being made on the charitable public.

It is also a matter of regret to find that in the hospitals where the charges for maintenance were held to be too high, no adequate reduction has yet been made. No doubt this matter will next year receive the close attention of the Distribution Committee.

With regard to King's College Hospital, I note with the utmost satisfaction its removal to South London. The importance of establishing in that vast population a large hospital, with a medical school, is known to all, and I am glad that the decision seems to have met with general approbation.

The amount allocated to the amalgamation of orthopædic hospitals is large, but I fully concur with the Distribution Committee in their view of the great importance of establishing in London one first-rate orthopædic hospital.

You will, I am sure, join with me in congratulating the League of Mercy on the steady progress made, and the increased aid given by it to the Fund.

It is a matter of satisfaction to find that the Trustees of the London Parochial Charities continue to entrust us with the administration of the £1,000 which they allot to Convalescent Homes. We are satisfied that in allocating these grants chiefly to convalescent homes connected with London hospitals we are acting in conformity with the views both of the supporters of the Fund and of the Trust.

The continued success of the Sunday and Saturday Funds proves that, as promised by the King, we have not trespassed upon their field of operations. The meeting of the Sunday Fund is to-morrow. We trust that the results may be in every way satisfactory, and we look for continued cooperation with the other Hospital Funds.

I have much pleasure in offering my congratulations to one of our honorary secretaries (Sir J. G. Craggs) upon the honour conferred upon him by his Majesty the patron of the Fund, in recognition of his valued services since the inauguration of the undertaking. (Hear, hear.)

I am directed by the King to express his high appreciation of the efficient manner in which the work of administrating the Fund has been conducted during the past year. His Majesty was especially pleased with the evident care and thought which had been bestowed upon the list of grants to hospitals, and with the judicious decisions come to by the distribution committee. I have now great pleasure in moving the adoption of the report.

The LORD MAYOR OF LONDON (Sir James Ritchie) said I rise with the greatest pleasure to second the adoption of this report which has been moved by his Royal Highness the President.

The DUKE OF FIFE, K.T.: I have much pleasure in supporting the adoption of the report. I only wish that the sum which this Fund has to distribute was larger. I think the Fund is admirably and economically managed, but of course the sum is very small when you think of what are the needs of the London hospitals. As I have said before (and I shall continue to do so) one of the great blots on London life is the very inadequate way in which people support the hospitals. In saying that I am not thinking so much of the rich and wealthy people as of the middle classes, the well-to-do workpeople, and tradespeople. I do not know what will happen, but I have a very strong view myself of what will be the end of the London hospitals. However, I shall not raise that question now, because it is a somewhat controversial one. I beg to second the adoption of the report, and I think we are all very much indebted to his Royal Highness for the interest he takes in this work. We ought to be very grateful to him for the amount of labour he has bestowed upon it. I hope some day his Royal Highness may be rewarded by seeing the annual income increased in a manner commensurate with his work and worthy of this great city—the largest city, I believe, in the civilised world.

The resolution was then put and carried unanimously.

The CHIEF RABBI (the Rev. Dr. Alder): May it please your Royal Highness, I beg to propose, in accordance with the recommendation of the Executive Committee that the cheques to the hospitals and convalescent homes shall be posted on the 22nd instant. I beg to congratulate your Royal Highness most sincerely on the results which have been achieved this year. It must be especially a source of extreme satisfaction that the forecast of H.M. the King has been so fully realised, and that not only has this Fund been so largely benefited by the public subscriptions, but also that the Mansion House Fund has not suffered. This is a stimulus and incentive to those contributing to the Fund to do better. This year on Hospital Sunday the weather was extremely inclement, yet a larger amount than ever was collected by the Sunday Fund. In moving this recommendation it must be a source of extreme gratification to us that such considerable sums have been voted by the Fund, and I know that to several of the hospitals no more welcome Christmas gift can be sent than the awards which will relieve them from their very great anxiety and their arduous responsibility."

Lord MONKSWELL, Chairman of the L.C.C., having seconded the proposition, it was agreed unanimously that all cheques should be posted on the 22nd inst.

The BISHOP OF LONDON: It is my pleasant duty to propose a vote of thanks to his Royal Highness for presiding on this occasion. What has been already said about the harmonious working of this and other funds is perfectly true, and I think we may trace our success to this and to the careful way in which the fund is administered. This is the right stimulus for hospital needs and hospital management. I very heartily move on behalf of everyone here a cordial vote of thanks to your Royal Highness for presiding here to-day. (Hear, hear.)

The Rev. J. GUINESS ROGERS, D.D., said: I have the greatest pleasure in seconding the vote of thanks to your Royal Highness. We all feel how much we owe to you for following the noble example of your Royal father in this matter. May I refer to a class who, especially in times of criticism, realise and appreciate your work still more—that is, the noble body of men who minister with patience and skill, and with success, to the wants of the sick throughout all our hospitals. It seems to me there are times when we ought to express our feelings in regard to such men, and on my own part I cannot express too strongly what I think in relation to the wide humanity as well as to the wonderful ability of the medical profession of this city. I am sure that everyone belonging to the hospitals feels the gratitude we owe to your Royal Highness. I second with great pleasure the motion that the thanks of this Council be given in the highest possible form to your Royal Highness for presiding over us on this occasion. (Hear, hear.)

The vote being unanimously carried, his ROYAL HIGHNESS said:—

I desire to thank you, My Lords and Gentlemen, for the kind manner in which this vote has been received. And I take this opportunity of expressing my gratitude to the Visiting Committee, Hospitals Distribution Committee, and Convalescent Homes Committee for the valuable time and labour which they have so readily given to the work of the Fund. I can only say there is a special satisfaction in discharging the duties of my office as President of this Fund, because I feel how loyally and efficiently I am supported by the gentlemen whom I have had the pleasure of meeting here to-day.

The proceedings then terminated.

